# Leveraging deep learning-based segmentation and contours-driven deformable registration for dose accumulation in abdominal structures

**DOI:** 10.3389/fonc.2022.1015608

**Published:** 2022-11-02

**Authors:** Molly M. McCulloch, Guillaume Cazoulat, Stina Svensson, Sergii Gryshkevych, Bastien Rigaud, Brian M. Anderson, Ezgi Kirimli, Brian De, Ryan T. Mathew, Mohamed Zaid, Dalia Elganainy, Christine B. Peterson, Peter Balter, Eugene J. Koay, Kristy K. Brock

**Affiliations:** ^1^ Department of Imaging Physics, The University of Texas MD Anderson Cancer Center, Houston, TX, United States; ^2^ RaySearch Laboratories, Stockholm, Sweden; ^3^ Department of Radiation Oncology, The University of Texas MD Anderson Cancer Center, Houston, TX, United States; ^4^ Department of Biostatistics, The University of Texas MD Anderson Cancer Center, Houston, TX, United States; ^5^ Department of Radiation Physics, The University of Texas MD Anderson Cancer Center, Houston, TX, United States

**Keywords:** dose accumulation, image-guided radiation therapy (IGRT), deformable image registration (DIR), liver cancer, GI toxicity

## Abstract

**Purpose:**

Discrepancies between planned and delivered dose to GI structures during radiation therapy (RT) of liver cancer may hamper the prediction of treatment outcomes. The purpose of this study is to develop a streamlined workflow for dose accumulation in a treatment planning system (TPS) during liver image-guided RT and to assess its accuracy when using different deformable image registration (DIR) algorithms.

**Materials and Methods:**

Fifty-six patients with primary and metastatic liver cancer treated with external beam radiotherapy guided by daily CT-on-rails (CTOR) were retrospectively analyzed. The liver, stomach and duodenum contours were auto-segmented on all planning CTs and daily CTORs using deep-learning methods. Dose accumulation was performed for each patient using scripting functionalities of the TPS and considering three available DIR algorithms based on: (i) image intensities only; (ii) intensities + contours; (iii) a biomechanical model (contours only). Planned and accumulated doses were converted to equivalent dose in 2Gy (EQD2) and normal tissue complication probabilities (NTCP) were calculated for the stomach and duodenum. Dosimetric indexes for the normal liver, GTV, stomach and duodenum and the NTCP values were exported from the TPS for analysis of the discrepancies between planned and the different accumulated doses.

**Results:**

Deep learning segmentation of the stomach and duodenum enabled considerable acceleration of the dose accumulation process for the 56 patients. Differences between accumulated and planned doses were analyzed considering the 3 DIR methods. For the normal liver, stomach and duodenum, the distribution of the 56 differences in maximum doses (D2%) presented a significantly higher variance when a contour-driven DIR method was used instead of the intensity only-based method. Comparing the two contour-driven DIR methods, differences in accumulated minimum doses (D98%) in the GTV were >2Gy for 15 (27%) of the patients. Considering accumulated dose instead of planned dose in standard NTCP models of the duodenum demonstrated a high sensitivity of the duodenum toxicity risk to these dose discrepancies, whereas smaller variations were observed for the stomach.

**Conclusion:**

This study demonstrated a successful implementation of an automatic workflow for dose accumulation during liver cancer RT in a commercial TPS. The use of contour-driven DIR methods led to larger discrepancies between planned and accumulated doses in comparison to using an intensity only based DIR method, suggesting a better capability of these approaches in estimating complex deformations of the GI organs.

## Introduction

Due to anatomical deformations during radiation therapy (RT), the delivered dose may differ from what was prescribed (1, 2), which may lead to an increased risk of toxicity for abdominal structures (3). Dose accumulation using deformable image registration (DIR) between daily volumetric imaging and the planning computed tomography (CT) scan allows, in theory, to better estimate the actual delivered dose and therefore the risk of toxicity. Unfortunately, abdominal structures prove challenging for DIR methods due to potential large deformation and change in contents such as gas or liquids between treatment fractions. Alignment of the stomach and duodenum appears especially challenging for traditional intensity-based DIR methods employed in the clinic, which may lead to inaccurate dose accumulation results (4).

The use of anatomical contours in biomechanical model-based DIR methods demonstrated to be promising for dose accumulation in the liver but was evaluated only for a limited number of patients (5, 6). To our knowledge, similar DIR studies have not been performed for the duodenum and stomach. A limitation of this contour-driven DIR approach is the requirement of consistent anatomical contours in the longitudinal images. Manual delineation of the organs can be prone to observer variability and is generally too time-consuming to be repeated on each daily image. However, with the recent advancements and availability of artificial intelligence (AI) tools, the possibility to perform rapid and accurate auto-segmentation should enable the use of contour-driven DIR for daily image-guided therapy (7, 8).

This study performs three innovative and clinically impactful studies on a large cohort of cases: i) develops and evaluates the use of deep-learning based segmentations of the stomach and duodenum, ii) evaluates the accuracy of dose accumulation when using different DIR algorithms (based only on image intensities or driven by contours) and iii) translates the impact of dose accumulation to normal tissue complication probabilities (NTCP) and compares between different algorithms and to planned dose.

## Materials and methods

### Patient data

Fifty-six patients diagnosed with primary liver cancer or metastatic disease in the liver treated with external beam radiation therapy (EBRT) between 2014 and 2019 at MD Anderson Cancer Center were retrospectively analyzed on an institutional review board (IRB) approved study. The 56 patients selected for this study were obtained from a cohort of 200+ patients enrolled on clinical protocols that included EBRT guided by daily CT-on-rails (CTOR) to treat primary cancers of the liver, based on availability of all data required for the study. In particular, only patients who had their stomach or duodenum fully encompassed by the CTOR Field-of-view (FOV) were included to allow the analysis of the dose accumulated in these two organs. Clinical data for the 56 patients can be viewed in **Table 1**. For all patients, planning CTs, daily CTORs, and planned dose distributions were imported into the research version of a commercial treatment planning system (TPS) RayStation (v10B, RaySearch Laboratories, Stockholm, Sweden). Planning CTs and planned dose distributions were acquired from the TPS Pinnacle (v9.10, Philips, Amsterdam, The Netherlands). CTOR, as well as their alignment to the planning CT, were imported directly from the radiation oncology clinic using an in-house Python-based script.

**Table 1:** Treatment parameters for patients in the dose accumulation cohort.

Characteristic	Value
Radiation therapy modality, conventional IMRT/SBRT [N]	51/5
Total dose (EQD2), range/median [Gy]	30-100/67.5
Total fractions, range/median [N]	4-28/15
Treatment duration, range/median [days]	4-38/21
Concurrent Chemotherapy [yes/no]	28/28
Liver Diagnosis, HCC/cholangiocarcinoma/metastasis/gallbladder [N]	22/18/15/1
Gender, male/female [N]	35/21
Age (at Tx start), range/median [years]	24-91/65

IMRT, intensity-modulated radiation therapy; SBRT, stereotactic body radiation therapy; EQD2, equivalent dose in 2Gy; N, number of patients; HCC, hepatocellular carcinoma.

### Tumor and normal tissue delineation

For all patients, clinical gross tumor volumes (GTV) contours on the planning CT were imported from Pinnacle. The whole liver was auto-contoured on the planning CT (including contrast-enhanced and non-contrast-enhanced) and CTOR using a previously validated deep learning 2D model (DeepLab V3+) (7). The stomach and duodenum were carefully re-delineated on each planning CT by the same physician-researcher (EK) under the guidance of a board-certified radiation oncologist (EJK) to ensure consistency in the definition of the contours between patients. As the stomach and duodenum are connected and the reproducibility of the interface of the organs can be challenging, the two organs were combined into a single structure to simplify the use of contour-based DIR methods and to avoid introducing irregular deformation due to the variability in defining the interface. To obtain a segmentation of this stomach and duodenum combination on all CTOR images, a standard 3D BasicUNet (9) implemented in the MONAI library (www.monai.io) was trained using the planning CT data only. The model was trained using Adam optimizer, cross-entropy loss function, and a 3D patch-based approach with patches of 128x128x64 voxels. Considering the relatively small size of the training data (56 cases), planning CTs from 56 additional abdominal CT images were collected and delineated specifically for the training of the UNet. The training, validation, and test cohorts consisted of 82, 20, and 10 patients, respectively. For the 10 test patients, the stomach and duodenum were contoured on one CT and one CTOR by EK. To evaluate the intra-observer variability, the same observer re-delineated these contours, without reference to the original contours, at least a week later. Contours were compared using Dice similarity coefficient (DSC). After inference on all CTOR images, the contours of the stomach and duodenum for the original 56 cases were reviewed. Minor manual editing was performed in 22% of cases and major editing in 15% of cases.

### Deformable image registration

Three DIR methods, available in the TPS, were used in the dose accumulation workflow to estimate the anatomical deformations between the CTOR images and planning CTs. The first method was ANACONDA (10) with no controlling regions of interest (ROI) (“Anaconda”), acting as a purely intensity-based DIR method. The second method was the same intensity-based method ANACONDA but also driven by boundary conditions on the liver and combined stomach and duodenum contours (“Anaconda_ROIs”). The third DIR method was a biomechanical model-based one, Morfeus (11, 12), driven only by the liver and combined stomach and duodenum contours (“Morfeus”), which has been extensively validated demonstrating voxel-based accuracy within the liver (12–14).

### Dose accumulation

Dose accumulation was conducted in the TPS based on each of the three DIR methods. Dose accumulation was implemented as part of the automated scripted workflow that first recalculates the doses on each daily image taking into account the associated couch shifts, applies the DIR algorithms to map the recalculated dose distribution on the planning CT, and finally sums the mapped doses. Accumulated doses based on each DIR method were compared to the planned dose. Accumulated and planned doses were measured for the normal liver (liver minus GTV), GTV, stomach, and duodenum. Maximum dose (D2%), average dose, and minimum dose (D98%) were calculated in the TPS and compared for each method. After dose accumulation in the TPS, all metrics were exported and processed for analysis using a prototype of RayIntelligence (RaySearch Laboratories). As fractionation schemes varied between patients in the study, doses were converted to equivalent dose in 2Gy (EQD2) using an α/β value of 2.5 Gy for the normal liver (15), 3.5 Gy for the stomach and duodenum (16), and 10 Gy as commonly used for the GTV.

### Normal tissue complication probability

NTCPs were calculated *via* scripting in the TPS for the stomach and duodenum using Lyman-Kutcher-Burman (LKB) models (17) for the planned and accumulated doses, using parameters proposed in previous studies: α/β = 2.5 Gy and n = 0.09 for the calculation of the generalized equivalent uniform dose (gEUD) (18); TD_50 = 24.6 Gy and m = .23 for the duodenum (19); TD_50 = 56 Gy and m = .21 for stomach (18).

### Statistical analysis

For the 56 patients, paired Student’s t-test were conducted to assess the significance of the differences between the mean of the three accumulated doses and planned dose. Separate analyses were conducted for the normal liver (liver minus GTV), GTV, stomach and duodenum. For the 3 DIR methods and 56 patients, the difference between accumulated and planned dose was calculated. The statistical significance of the mean and variance difference between the three dose difference distributions were assessed with paired Student’s t-test and F-test, respectively. Because of the multiple comparisons, all p-values were adjusted with the Bonferroni method. Differences in means and variances were considered statistically significant for adjusted p <0.05. A similar analysis was conducted for the NTCP values of the duodenum and stomach.

## Results

### Stomach and duodenum auto-segmentation

**Table 2** shows the DSC between deep learning-based and physician-drawn segmentation of combined stomach and duodenum for the ten validation patients on CT and CTOR; DSC for intra-observer variability; and using Anaconda, DSC between physician drawn and deformed contours.

**Table 2:** Dice similarity coefficients (DSC) for the combination of stomach and duodenum in 10 test pairs of CT and CTOR from different patients.

	Manual *vs* Auto-segmentation		Intra-observer variability		Manual *vs* DIR
Patient	CT	CTOR	CT	CTOR	propagation
1	0.87	0.94	0.87	0.94	0.75
2	0.88	0.92	0.89	0.93	0.73
3	0.88	0.87	0.89	0.94	0.56
4	0.72	0.87	0.88	0.89	0.93
5	0.92	0.92	0.90	0.92	0.84
6	0.91	0.93	0.89	0.92	0.83
7	0.93	0.94	0.91	0.94	0.81
8	0.93	0.92	0.93	0.91	0.97
9	0.88	0.89	0.92	0.88	0.59
10	0.92	0.93	0.95	0.93	0.89
Mean	0.88	0.91	0.90	0.92	0.79
SD	0.06	0.02	0.02	0.02	0.13
Min	0.72	0.87	0.87	0.88	0.56
Max	0.93	0.94	0.95	0.94	0.97

**Figure 1:**
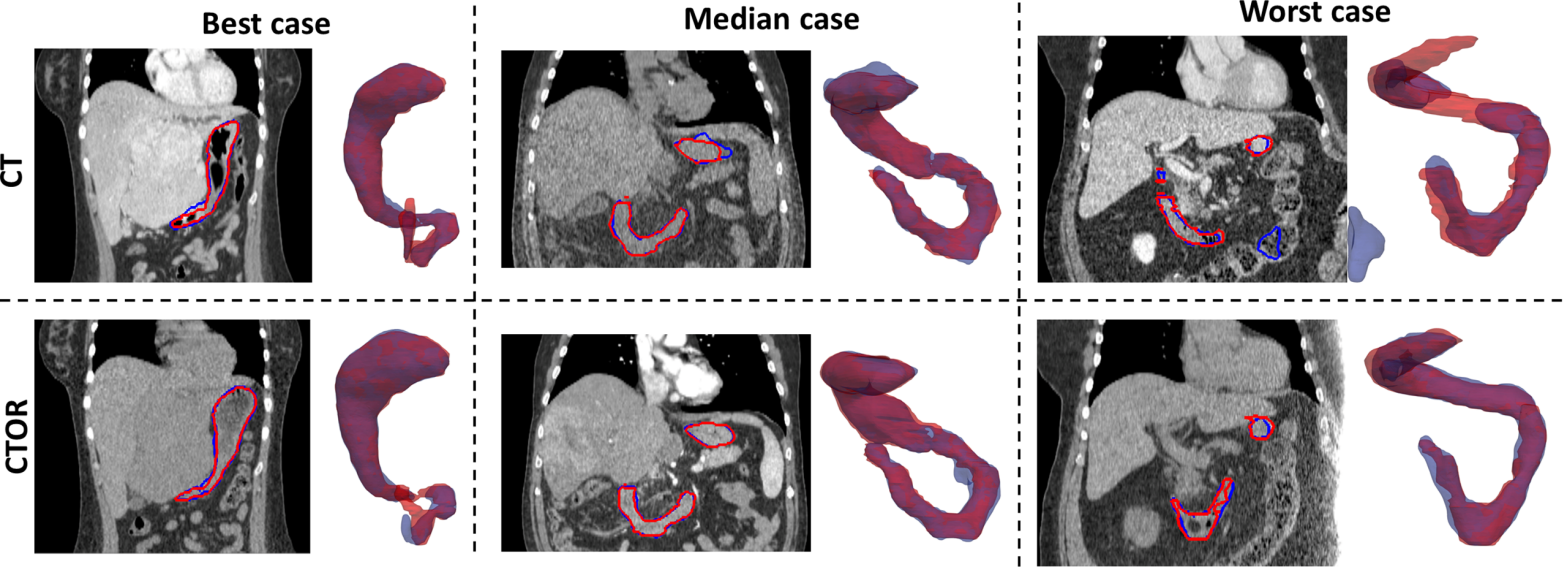
Representation for 3 test cases of manually-drawn (in red) and auto-segmented (in blue) contours of the stomach and duodenum combination on transversal slices of the images and 3D perspective (posterior to anterior direction). Top row: CT scans; Bottom row: CTOR scans.

Means were not statistically significant when comparing DSC between deep learning-based versus intra-observer variability (p>0.05; Student’s 2-tailed paired t-test). The use of auto-segmentation on CTOR led to a significantly higher mean DSC than when using the DIR method to map the contour from the CT (0.91 *vs* 0.79, p <0.05). The relatively low DSCs between DIR propagated contours, on average 0.79, illustrate the poor performance of this intensity-only DIR method in aligning these two organs. **Figure 1** represents an example of auto-segmented contours versus the physician drawn ground-truth for the best, median and worst cases (patients 10, 5 and 4, respectively, according to the average of DSC on CT and CTOR).

### Normal liver EQD2

The distributions of the planned and accumulated doses as well as differences between accumulated and planned doses are represented in **Figure 1**. For the differences between accumulated and planned dose, no significant differences were observed for average and maximum doses. However, the average dose difference between accumulated and planned dose was >1Gy for 8 (14%), 22 (39%) and 20 (36%) patients for Anaconda, Anaconda_ROIs and Morfeus, respectively. The maximum dose (D2%) difference between accumulated and planned dose was >5Gy for 10 (18%), 16 (29%) and 14 (25%) patients for Anaconda, Anaconda_ROIs and Morfeus, respectively. For the two contour-driven DIR methods, Anaconda_ROIs and Morfeus: the differences between planned and accumulated maximum dose (D2%) had a significantly higher variance compared to Anaconda, with ratio of variances of 2.1 (p <0.05) and 2.7 (p <0.01), respectively.

### GTV EQD2

**Figure 2:**
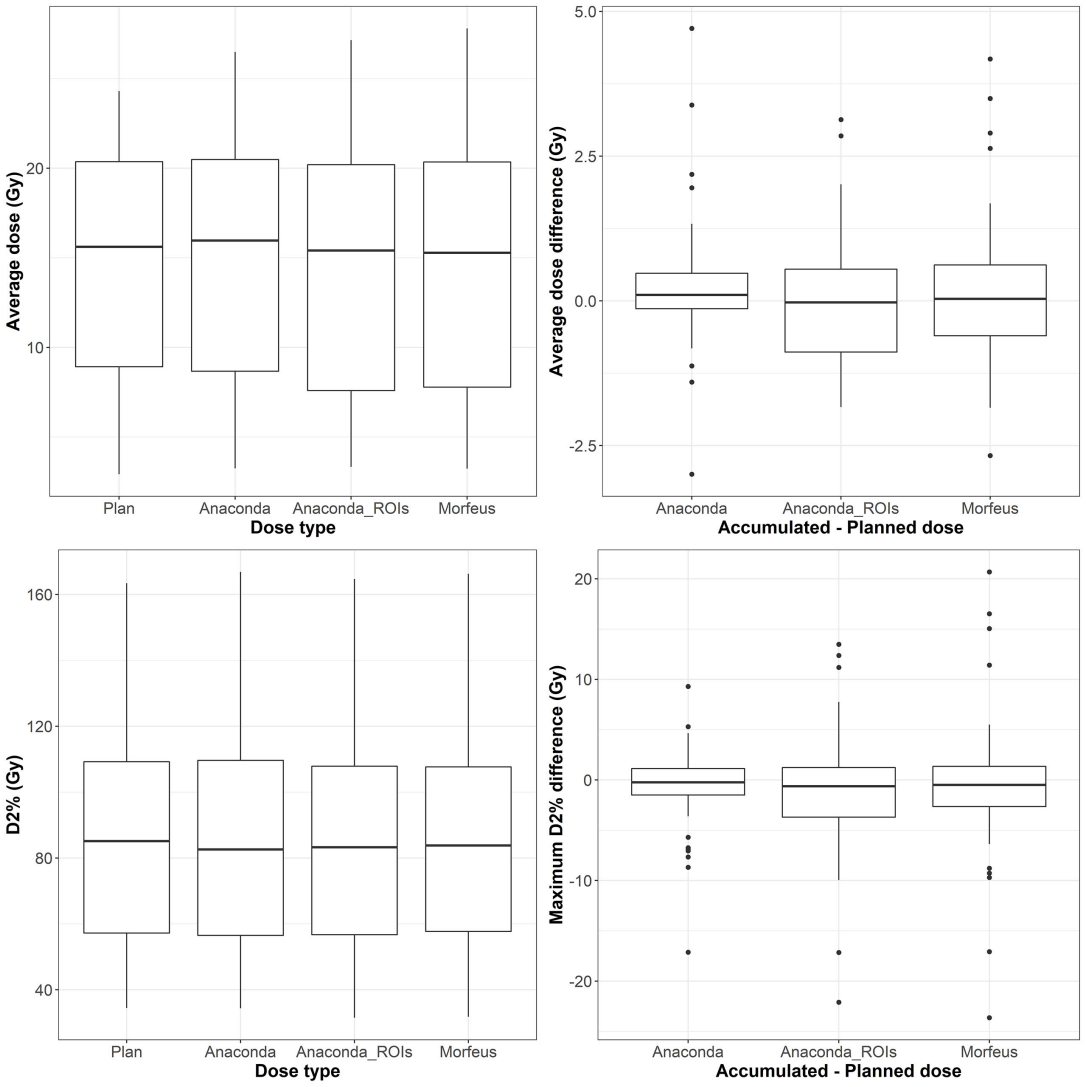
Left: average (top row) and maximum (bottow row) planned and accumulated normal liver EQD2 (Gy). Right: difference between accumulated and planned doses.

The distributions of the planned and accumulated doses as well as differences between accumulated and planned doses for the GTV are represented in **Figure 2**. For the differences between accumulated and planned dose, the mean values of the average dose were statistically different between Anaconda and Anaconda_ROIs (p <0.01), between Anaconda and Morfeus (p <0.01) and between Anaconda_ROIs and Morfeus (p <0.05). The average dose difference between accumulated and planned dose was >1Gy for 25 (45%), 32 (57%) and 33 (59%) patients for Anaconda, Anaconda_ROIs and Morfeus, respectively. The mean values of the minimum dose differences were statistically different between Anaconda and Anaconda_ROIs (p <0.01) and between Anaconda and Morfeus (p <0.01). The difference of minimum dose between accumulated and planned dose was >5Gy for 6 (11%), 28 (50%) and 24 (43%) patients for Anaconda, Anaconda_ROIs and Morfeus, respectively. For the two contour-driven DIR methods, Anaconda_ROIs and Morfeus: the differences between planned and accumulated average dose had a significantly higher variance compared to Anaconda, with ratio of variances of 3.7 (p <0.01) and 2.1 (p <0.05), respectively; the differences between planned and accumulated minimum dose had a significantly higher variance compared to Anaconda, with ratio of variances of 10.4 (p <0.01) and 12.5 (p <0.01), respectively.

### Duodenum EQD2

**Figure 3:**
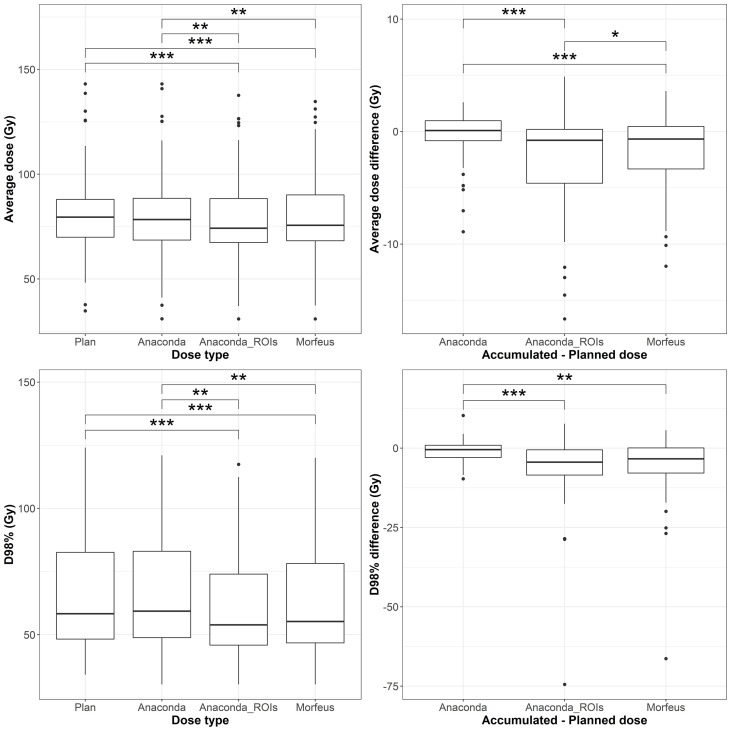
Left: average (top row) and maximum (bottow row) planned and accumulated GTV EQD2 (Gy). Right: difference between accumulated and planned doses. Statistically significant differences between means are indicated according to Bonferroni-adjusted p-values (*p <0.05; **p <0.01; ***p <0.001).

The distributions of the planned and accumulated doses as well as differences between accumulated and planned doses for the duodenum are represented in **Figure 3**. For the differences between accumulated and planned dose: the mean values of the average dose were statistically different between Anaconda and Morfeus (p <0.05) and between Anaconda and Anaconda_ROIs (p <0.05); the mean values of the maximum doses were statistically different between Anaconda and Anaconda_ROIs (p <0.01) and between Anaconda and Morfeus (p <0.01). The average dose difference between accumulated and planned dose was >1Gy for 9 (16%), 14 (25%) and 14 (25%) patients for Anaconda, Anaconda_ROIs and Morfeus, respectively. The maximum dose difference between accumulated and planned dose was >5Gy for 2 (4%), 10 (18%) and 12 (21%) patients for Anaconda, Anaconda_ROIs and Morfeus, respectively. For the differences in average dose, both Anaconda_ROIs and Morfeus had a significantly higher variance compared to Anaconda, with ratio of variances of 5.1 (p <0.01) and 5.5 (p <0.01), respectively. For the differences in maximum doses, Anaconda_ROIs and Morfeus also yielded to a significantly higher variance compared to Anaconda, with ratio of variances of 5.4 (p <0.01) and 3.7 (p <0.01), respectively.

### Stomach EQD2

**Figure 4:**
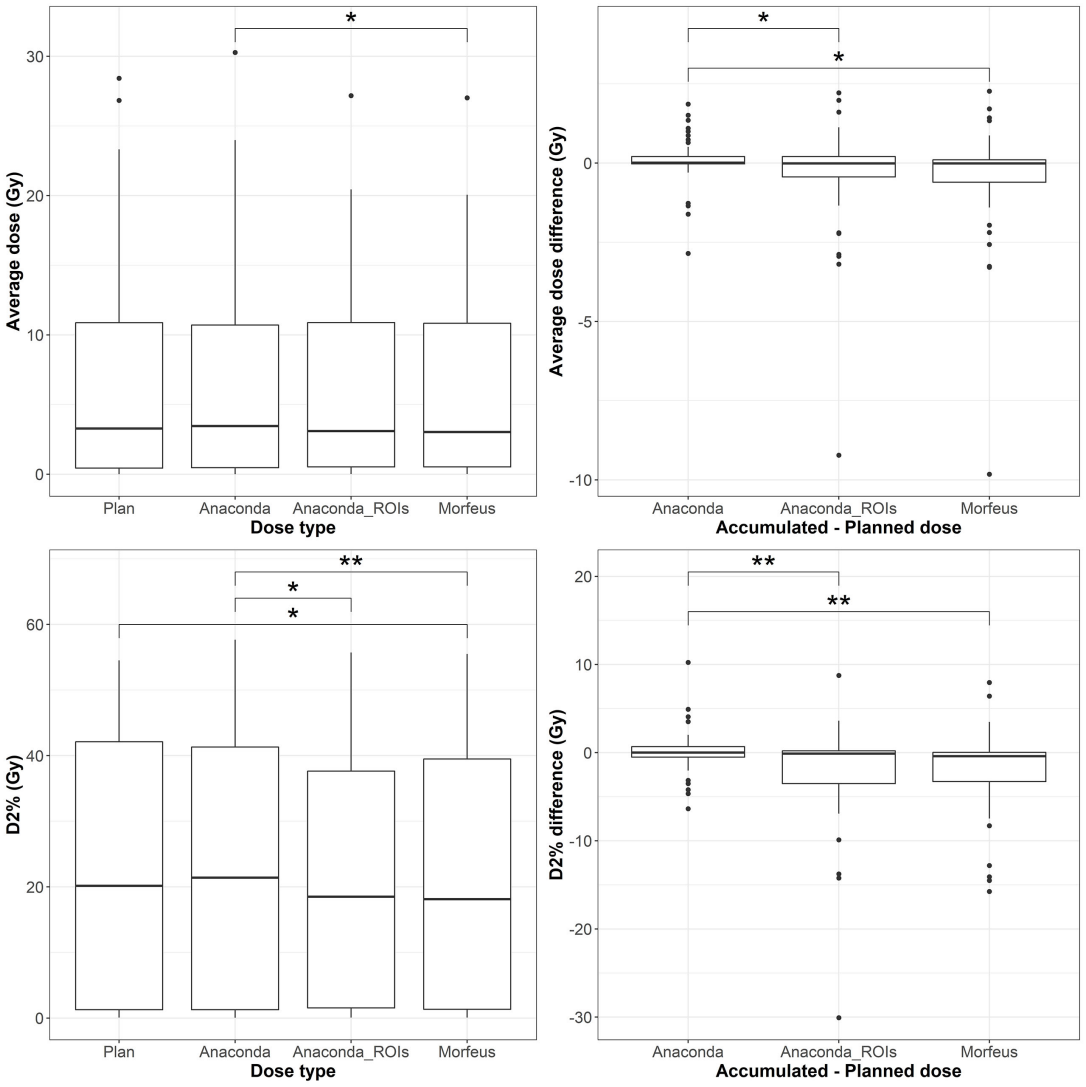
Left: average (top row) and maximum (bottow row) planned and accumulated Duodenum EQD2 (Gy). Right: difference between accumulated and planned doses. Statistically significant differences between means are indicated according to Bonferroni-adjusted p-values (*p <0.05; **p <0.01).

The distributions of the planned and accumulated doses as well as differences between accumulated and planned doses for the stomach are represented in **Figure 4**. For the differences between accumulated and planned dose, the mean values of the maximum dose were statistically different between Anaconda and Anaconda_ROIs (p <0.01), between Anaconda and Morfeus (p <0.001), and Anaconda_ROIs and Morfeus (p <0.05). The average dose difference between accumulated and planned dose was >1Gy for 5 (9%), 7 (13%) and 8 (14%) patients for Anaconda, Anaconda_ROIs and Morfeus, respectively. The maximum dose difference between accumulated and planned dose was >5Gy for 2 (4%), 5 (9%) and 5 (9%) patients for Anaconda, Anaconda_ROIs and Morfeus, respectively. For the differences in average dose, both Anaconda_ROIs and Morfeus had a significantly higher variance compared to Anaconda, with ratio of variances of 2.1 (p <0.05) and 2.2 (p <0.05), respectively. For the differences in maximum doses, Anaconda_ROIs and Morfeus also yielded to a significantly higher variance compared to Anaconda, with ratio of variances of 2.1 (p <0.05) and 2.3 (p <0.01), respectively.

### Accumulated EQD2 differences between contour-driven DIR methods

Except for the stomach maximum dose (D2%), no significant mean differences were observed between the accumulated EQD2 distributions obtained with Anaconda_ROIs and Morfeus. However, substantial differences were observed for some patients. Differences in average accumulated EQD2 between Anaconda_ROIs and Morfeus were >1Gy for 4 (7%) and 16 (29%) of the normal livers and GTVs, respectively. No differences were found for the duodenums and stomachs. Differences in maximum doses (D2%) were >2Gy for 11 (20%), 5 (9%), and 0 of the normal livers, duodenums and stomachs, respectively. Differences in minimum doses (D98%) were >2Gy for 15 (27%) of the GTVs.

### Duodenum and stomach NTCPs

**Figure 5:**
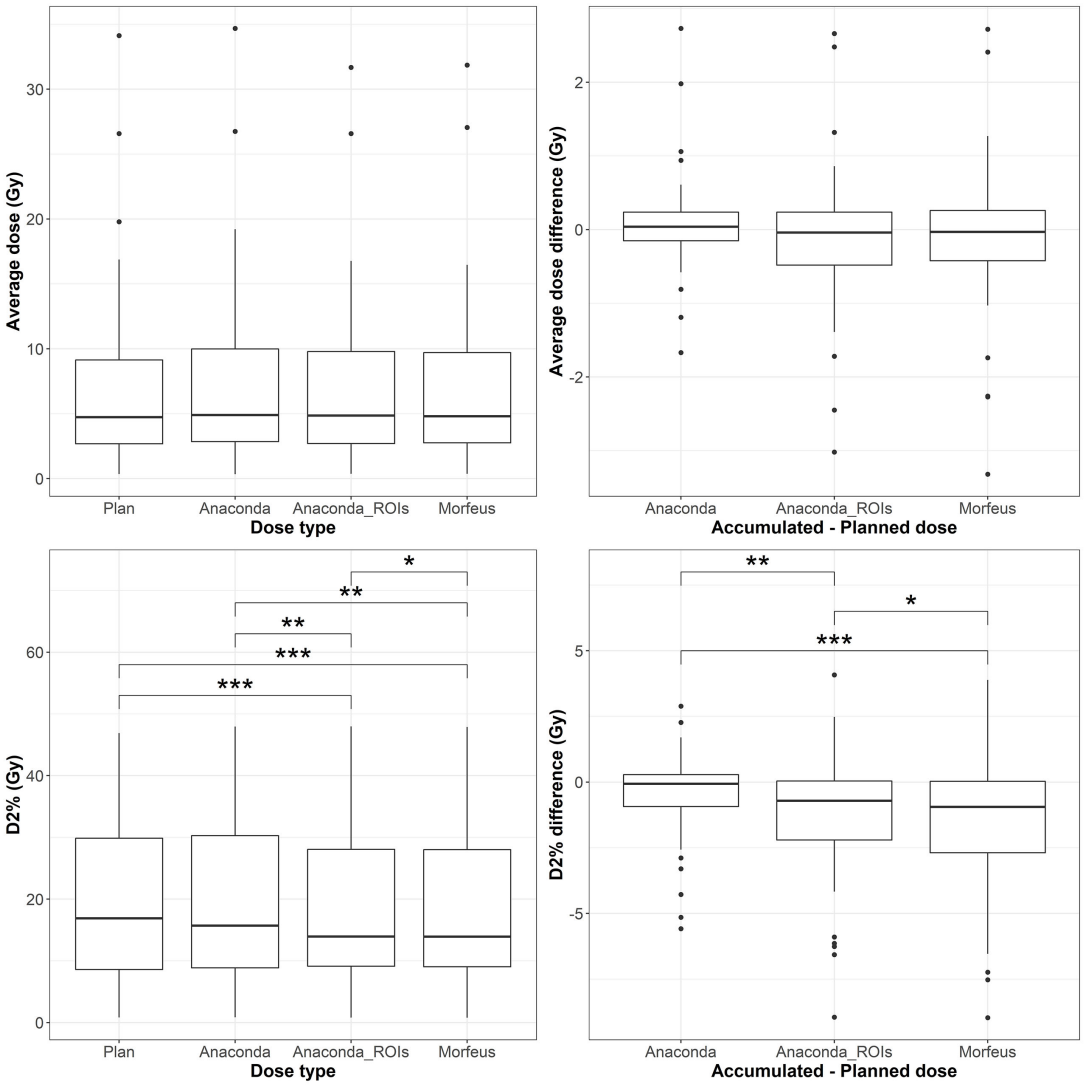
Left: average (top row) and maximum (bottow row) planned and accumulated Stomach EQD2 (Gy). Right: difference between accumulated and planned doses. Statistically significant differences between means are indicated according to Bonferroni-adjusted p-values (*p <0.05; **p <0.01; ***p <0.001).

**Figure 6:**
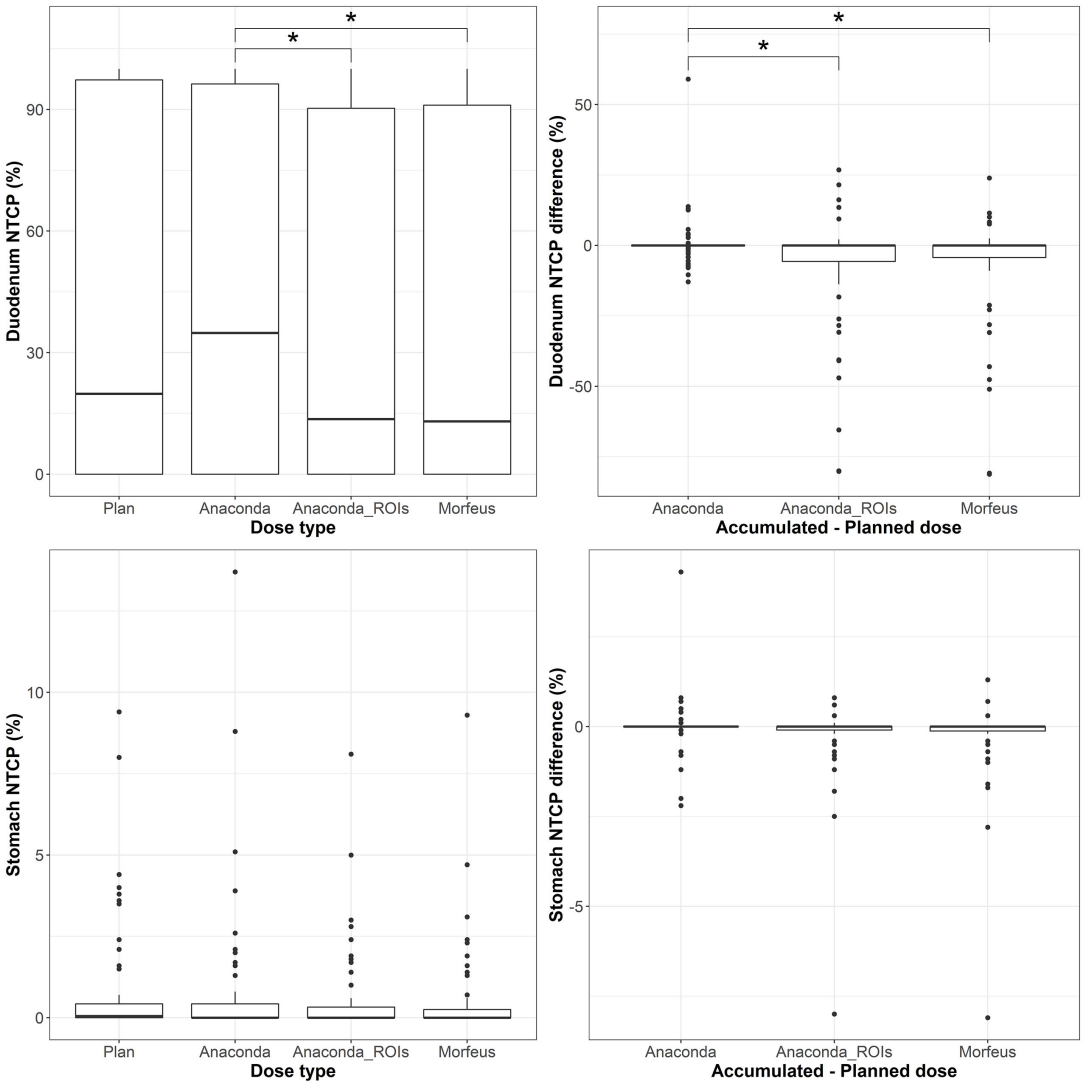
Top row: duodenum; Bottom row: stomach; Left: NTCPs; Right: NTCP differences between accumulated and planned dose. Statistically significant differences between means are indicated according to Bonferroni-adjusted p-values (*p <0.05).

The distributions of the NTCP based on planned and accumulated doses and differences between accumulated and planned NTCP are represented in **Figure 5** for the duodenum and in **Figure 6** for the stomach. With the model parameters considered, the nominal NTCP varied between 0 and 100% for the duodenum and between 0 and 9.4% for the stomach. Depending on the DIR method, the differences in NTCP using accumulated or planned dose for calculation ranged from -81.2% to +59% for the duodenum and only from -8.1% to +4.3% for the stomach. For the duodenum, the mean NTCP obtained using either of the contour-driven methods was significantly different from the mean obtained using Anaconda (p <0.05). The NTCP differences obtained with contour-driven methods also presented a significantly higher variance in comparison to the NTCP differences obtained using Anaconda (p <0.01), with ratio of variances of 4.7 and 5.4 for Morfeus and Anaconda_ROIs, respectively. No statistical difference was observed for the mean stomach NCTP, but both contour-driven methods led to significantly higher variances in NTCP differences than when considering Anaconda (p <0.01), with ratio of variances of 2.5 and 2.3 for Morfeus and Anaconda_ROIs, respectively.

## Discussion

While tools to accumulate dose from daily imaging during RT are available today in most treatment planning system, the results can vary widely due to DIR uncertainties. To prevent the estimation of unrealistic deformation between longitudinal images, standard DIR algorithms based on intensity information alone generally tend to be highly constrained in degrees of freedom. These constraints may prevent accurate alignment of organs presenting with complex deformations independently of the surrounding anatomy, such as the stomach or duodenum. In this study, the intensity-based DIR method (Anaconda) seemed to underestimate the discrepancies between delivered and planned doses for the stomach and duodenum in comparison to the contour-driven methods (Anaconda_ROIs and Morfeus), as suggested by the low DSC observed after contour propagation between ten pairs of CT and CTOR scans and the significant differences in ratios of variance for average and maximum dose. Considering accumulated dose instead of planned dose in standard NTCP models of the duodenum demonstrated a high sensitivity of the duodenum toxicity risk to these dose discrepancies. Much smaller variations were observed for the stomach, which initially presented with very low risk of toxicity in this study to its increased distance to the prescribed dose.

The higher contrast, larger volume and moderate deformations of the liver in comparison to the other organs, allowed the intensity-based method to successfully align the liver boundaries in most of the cases, based on visual inspection. However, in some cases, obvious misalignment of the liver boundaries could still be observed after intensity-based DIR leading to substantial differences in average accumulated doses compared to the driven-contour methods. Considering the contour-driven DIR methods, the difference between accumulated and planned mean dose to the normal liver was above 1Gy for about one third of the patients (36% and 39% for Morfeus and Anaconda_ROIs, respectively). As previous studies observed a correlation between mean dose to the liver (20), or V30 (21), and radiation-induced liver disease (RILD), the use of such contour-driven methods appears necessary for the development of new liver toxicity prediction models based on accumulated dose.

The choice of the DIR approach for the liver is also important for the estimation of the accumulated dose in the GTV. As no contrast usually exists inside the liver on the daily CTORs, the GTV registration is entirely based on the global liver registration. The variances of the accumulated minimum and average doses in the GTV were significantly higher when using a contour-driven method than when using the intensity-only method, suggesting the latter may underperform at detecting under-dosage of the GTV. While no significant differences in mean were observed between the dose distributions for the two contours-driven methods, the differences in minimum dose (D98%) was >2Gy for 27% of the patients. These differences combined with prior evidence that biomechanical model-based DIR has a higher accuracy compared to intensity-based DIR methods, suggests that the use of biomechanical-base DIR is important for dose accumulation in organs where the tumor is not directly visible on the daily image used for dose accumulation (22).

This study compared DIR methods using only accumulated dose indexes such as average, minimum and maximum doses. These global indexes can be insensitive to local variations of the estimated displacement vector field (23). Other applications requiring accurate DIR at the voxel level would require further evaluation of local accuracy of the DIR methods, for example for the analysis of the voxel-wise correlation between accumulated dose and change in functional imaging such as liver perfusion maps (24). However, the lack of contrast on the CTOR images did not allow the extraction of anatomical landmarks, for example vessel bifurcations inside the liver (25), to measure the local DIR accuracy. Evaluation of the DIR accuracy in matching the stomach and duodenum is particularly challenging due to the lack of anatomical landmarks on their smooth surface. Ultimately, the dose accumulated with one DIR method or the other could show a higher correlation with toxicity outcomes, suggesting which one was the most accurate.

A fully automatic workflow, using scripting in the TPS, has been developed, allowing the analysis of accumulated dose for a substantial number of patients. While most clinical TPS now offer a toolbox to perform dose accumulation, the process still usually requires numerous manual interactions, including rigid alignment of the fraction images according to the patient positioning under the linear accelerator, calculating the dose on the daily image, delineating organs if contour-driven DIR is desired, running the DIR, applying the resulting deformation to the recalculated dose, and finally, assessing the discrepancies between planned and accumulated dose indexes. Repeating this process for each fraction and eventually to assess the results using different DIR methods can be overly time-consuming, which may explain the limited number of studies reporting dose accumulation. The auto-segmentation did lack sufficient accuracy on all cases requiring manual editing, however it still enabled considerable acceleration in delineation of abdominal structures on all fraction images, which would otherwise require several hours. However, as more patients are analyzed in future work and contours eventually edited, the deep-learning model will be re-trained with these additional data, which should improve the robustness of the auto-segmentation. Clinical translation of these findings includes improved DIR-driven dose accumulation methods along with demonstrated impact on the improvement of toxicity prediction modeling. Studies to evaluate if the correlation between toxicity and accumulated dose is stronger than when considering planned dose only are underway. To minimize the effects of confounding factors of those toxicities, larger cohorts of patients can be included using the proposed automatic workflow directly integrated in a TPS. These advancements will ultimately help to optimize treatment planning for oncology patients and allow radiation oncologists to give a higher dose directly to the tumor and lower dose to surrounding normal tissue, sparing unnecessary long-term damage.

## Conclusions

This study has demonstrated the successful implementation of a fully automatic workflow for dose accumulation during liver cancer radiotherapy in a commercial TPS, its impact on the calculation of delivered dose and the translation to outcomes prediction. As dose accumulation methods suggest lower doses of radiation to the GTV than intended could occur, there is potential for decreased tumor control and a higher chance of recurrence when image-guided radiation therapy is not utilized. The differences seen between planned and accumulated dose suggest that dose accumulation should be used during RT for patients with liver disease. Finally, our work highlights the significance of recognizing the variance between different dose accumulation methods and emphasizes the need for advanced toxicity models to investigate the correlations between outcomes and true delivered dose, in order to assess the most accurate dose accumulation technique.

## Data availability statement

The data analyzed in this study is subject to the following licenses/restrictions: Request for access to data will be reviewed by institutional IRB. Requests to access these datasets should be directed to gcazoulat@mdanderson.org.

## Ethics statement

The studies involving human participants were reviewed and approved by MD Anderson Cancer Center IRBs. No written informed consent was required from the participants as this was a retrospective study, and images and radiation treatment plans were obtained retrospectively under an IRB-approved protocol.

## Author contributions

Study concepts: MM, GC, EJK, and KB. Study design: MM, GC, EJK, and KB. Data acquisition: MM, BD, EK, RM, MZ, DE, and PB. Quality control of data and algorithms: MM, GC, SS, SG, BR, and BA. Data analysis and interpretation: MM, GC, CP, and KB. Manuscript editing: MM, GC, and KB. All authors contributed to the article and approved the submitted version.

## Funding

Research reported in this publication was supported in part by the Helen Black Image Guided Fund, in part by resources of the Image Guided Cancer Therapy Research Program at The University of Texas MD Anderson Cancer Center, in part by the National Cancer Institute of the National Institutes of Health under award number 1R01CA221971, in part by RaySearch Laboratories AB and University of Texas MD Anderson Cancer Center through a Co-Development and Collaboration Agreement, in part by the National Institutes of Health/NCI under award number P30CA016672. Dr. EK was supported by the Andrew Sabin Family Fellowship, the Sheikh Ahmed Center for Pancreatic Cancer Research, institutional funds from The University of Texas MD Anderson Cancer Center, the Khalifa Foundation, equipment support by GE Healthcare and the Center of Advanced Biomedical Imaging, Cancer Center Support (Core) Grant CA016672, and by NIH (5P50CA217674-2, U54CA210181, U54CA143837, U01CA200468, U01CA196403, U01CA214263, R01CA221971, R01CA248917, and R01CA218004).

## Conflict of interest

Dr. PB reports support for sponsored research from RaySearch Laboratories and Varian. Dr. KB reports Licensing agreement with RaySearch Laboratories for Deformable Image Registration technologies, participation on RaySearch Clinical Advisory Board.

The remaining authors declare that the research was conducted in the absence of any commercial or financial relationships that could be construed as a potential conflict of interest.

## Publisher’s note

All claims expressed in this article are solely those of the authors and do not necessarily represent those of their affiliated organizations, or those of the publisher, the editors and the reviewers. Any product that may be evaluated in this article, or claim that may be made by its manufacturer, is not guaranteed or endorsed by the publisher.
